# Paget’s disease of the male breast: a case report

**DOI:** 10.1186/s40792-015-0105-6

**Published:** 2015-10-15

**Authors:** Masayuki Akita, Nobuya Kusunoki, Takahiro Nakajima, Shiro Takase, Yoko Maekawa, Kazuyoshi Kajimoto, Masakazu Ohno

**Affiliations:** Department of Surgery, Hyogo Prefectural Kaibara Hospital, 5208-1 Kaibara, Kaibara-cho, Tamba, Japan; Division of Hepato-Biliary-Pancreatic Surgery, Department of Surgery, Kobe University Graduate School of Medicine, 7-5-2 Kusunoki-cho, Chuo-ku, Kobe, Japan; Department of Surgery, National Hospital Organization Kobe Medical Center, 3-1-1 Nishiochiai, Suma-ku, Kobe, Japan; Department of Pathology, Hyogo Cancer Center, 13-70, Kitaoji-cho, Akashi, Japan

**Keywords:** Male breast cancer, Mammary Paget’s disease, Sentinel lymph node biopsy, Mapping biopsy

## Abstract

The patient was a 91-year-old man with change in nipple appearance, itching and redness, and a palpable breast mass. At presentation, mammary Paget’s disease (PD) was clinically suspected. Skin biopsy was performed and showed epidermis invaded by Paget cells, characterized by hyperchromatic nuclei and abundant pale-staining cytoplasm. Computed tomography and mammary ultrasonography confirmed the absence of an underlying invasive carcinoma, and the patient underwent right mastectomy and sentinel lymph node biopsy (SLNB). Both sentinel lymph nodes were found to be negative perioperatively, and further axillary dissection was not performed. Pathological results revealed no malignancy under the nipple, yet the Paget cells were more widely spread than expected. The patient was followed up without the need of postoperative chemotherapy. Male mammary PD is an extremely rare breast cancer, and there is no standard preoperative assessment or operative procedure. Mammography is many times unable to detect possible underlying breast carcinoma in female patients with mammary PD, and previous studies have reported that the detection rate was less than 50 %. However, some researchers reported that magnetic resonance imaging (MRI) might be more detectable to confirm the extent of the cancer. The extent of the skin change around the nipple is often different from the actual perimeter of Paget cells. In extra-mammary PD, mapping biopsy is known to be useful to determine areas free of cancer. The benefits of SLNB have also been demonstrated for the management of less invasive breast cancers, and previous reports have shown that the use of SLNB is reasonable for treatment of mammary PD without underlying invasive cancer. MRI, mapping biopsy, and SLNB are all less invasive procedures and thus may be suitable for treatment of male mammary PD.

## Background

Mammary Paget’s disease (PD) is rare and comprises about 1 % of all breast cancers. Male breast cancer is also uncommon, representing approximately 1.0 % of all breast malignancies. Thus, male mammary PD of the breast is an extremely rare occurrence [1, 2]. Between 1980 and 2015, eight case reports were identified from the MEDLINE by keywords, “mammary Paget disease, man” (Table 1). Most patients with mammary PD initially note the presence of redness, erosion, or pruritus of the nipple and are diagnosed by skin biopsy or nipple discharge cytology, which is characterized by malignant glandular cells with clear cytoplasm and eccentric, hyperchromatic nuclei within the epidermis. PD of the breast is accompanied by invasive or noninvasive underlying carcinomas in 84~94 % of the cases [3–5], and the prognosis of mammary PD is correlated with the stage of the underlying carcinoma. From the preoperative assessment of the extent of the spread of the tumor, surgeons decide the operative management of the mammary PD, mastectomy with or without an axial lymph node dissection. This report presents a preoperative and surgical strategy for male mammary PD.

**Table 1:** Mammary Paget’s disease of the man in the literature

Patient no.	Author	Age	Symptoms	Palpable mass	Treatment	Axillary metastasis	Follow-up
1	Lancer HA [9]	81	Irritation, redness	−	Mastectomy	−	Disease free 5 months
2	Serour F [6]	73	Lump, eczema	+	Mastectomy irradiation	+	Disease free 8 years
3	O’Sullivan ST [1]	72	Erythema, eczema	+	Simple mastectomy	N/A	alive 9 months
4	Hayes R [7]	65	Bloody discharge, ulceration	−	Mastectomy adjuvant chemotherapy	+	Disease free 5 months
5	Nakamura S [8]	83	Bloody discharge, pigmented	−	Simple mastectomy	N/A	Disease free 9 months
6	Bernardi M [19]	52	Discolored, pruritus	−	Failed to return for follow-up	N/A	N/A
7	Ucar AE [10]	74	Bilateral excoriation	rt + lt −	Mastectomy adjuvant chemo-radiotherapy	−	N/A
8	Harroudi T [2]	61	Pruritus, erythema	+	Mastectomy adjuvant chemo-radiotherapy	−	Disease free 2 years
9	Current case	91	Itching, redness	−	Mastectomy SLNB	−	Disease free 1 years

## Case presentation

**Fig. 1:**
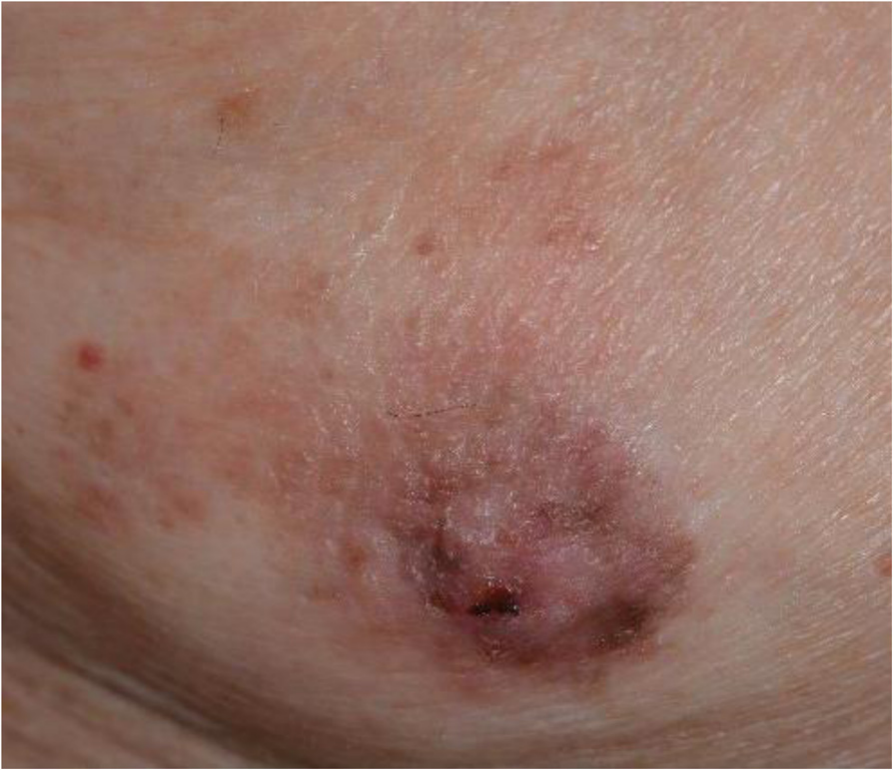
Right breast at initial presentation. Erosion and redness of the nipple were noted, and lateral accretion of Paget’s disease was suspected. A 1.0-cm movable lump was identified in the breast

**Fig. 2:**
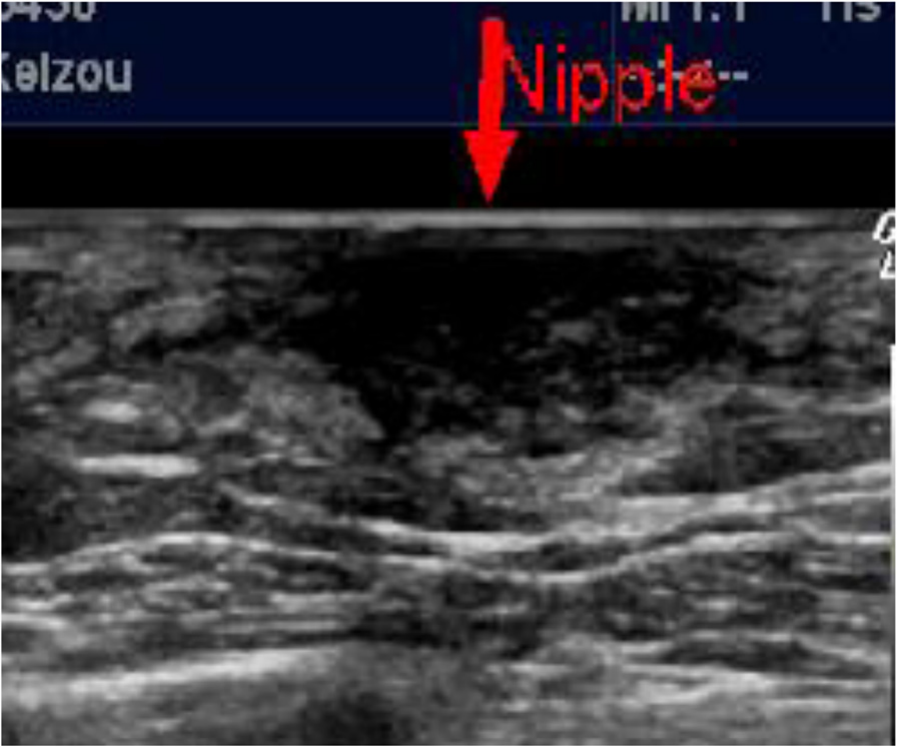
Mammary ultrasonography. A poorly marginated and low echoic area was detected under the nipple. The palpable mass was diagnosed as gynecomastia

**Fig. 3:**
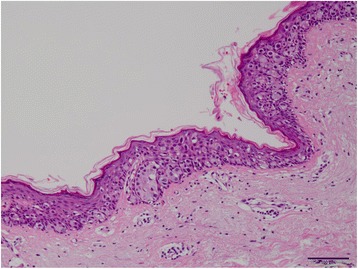
Histopathological findings of the resected specimen (Hematoxylin-eosin stain, ×100). Paget’s cells, which were characterized by clear cytoplasm and hyperchromatic nuclei, were detected within the epidermis. Deep infiltration was not observed

**Fig. 4:**
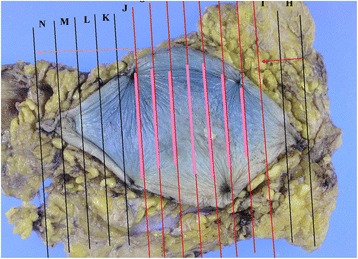
Excised specimen from the mastectomy. A histopathological cancer map of the mastectomy specimen with the *bold pink lines* showing the detected malignant area. This area was not consistent with the preoperative assessment. Negative margins were confirmed

The patient was a 91-year-old man with no family history of testicle, breast, and ovarian disease, who noted itchiness and redness around his right nipple with a palpable mass underneath (Fig. 1). Physical examination revealed a 1.0-cm movable mass underneath the nipple with no axial lymphadenopathy. The full-thickness skin biopsy identified Paget cells in the epidermis, and immunohistochemistry showed these cells were stained strongly for cytokeratin 7, cytokeratin 20, and with alcian blue and was negative for gross cystic disease fluid protein 15; these results were consistent with PD. The evaluation of the overexpression of human EGFR-related 2 protein showed weakly positive cells (score2: equivocal). The mass under his nipple was diagnosed as gynecomastia by mammary ultrasonography (Fig. 2). Blood examination showed normal tumor markers (squamous cell carcinoma, 1.1 ng/dl, carcinoembryonic antigen, 5.2 ng/dl, carbohydrate antigen 15–3, 9.6 ng/dl) and normal liver function. Computed tomography confirmed absence of an invasive tumor and distant metastases. The patient underwent right total mastectomy and right sentinel lymph node biopsy. Two sentinel lymph nodes were removed, and due to no metastases in the axilla, axillary lymph node dissection was not performed. No underlying carcinoma was found in the resected specimen, and Paget cells were found to be spread more extensively to the skin than expected (Fig 3), although the resected margin was negative (Fig. 4). Due to the patient’s age and no invasive nest other than Paget infiltration to the skin, the patient was followed up without the need of postoperative adjuvant therapy.

## Conclusions

Between 1980 and 2015, eight case reports were identified from the MEDLINE (Table 1). The age of patients varies from 52 to 91 years. Almost all patients presented to the outpatient department with a chief complaint of skin changes around the nipple. Some also noticed the nipple bloody discharge, and only one did an underlying palpable mass as a primary symptom [6–8]. In physical examinations, none of them had axillary lymphadenopathy, but at least two patients had axillary metastases in the resected specimen [6, 7]. The rate of patients without underlying carcinomas may be higher than females. However, much a short follow-up period, none of the patients have died of the recurrence of the mammary PD. Because survival depends on the stage of the underlying carcinoma, the low rate of the underlying tumor seems to result in good prognosis [2, 6, 9, 10]. The effectiveness of axillary lymph nodes dissection, irradiation, or adjuvant chemotherapy for male mammary PD is still uncertain, but as with female mammary PD, these options should be considered on the basis of the stage of cancer progression.

In clinically suspected cases of mammary PD, full-thickness skin biopsy is used to pathologically obtain a diagnosis as mammary PD, and mammography and mammary ultrasonography are used to identify the presence of an underlying carcinoma. Female mammary PD patients (84~94 %) had an underlying invasive or noninvasive carcinoma [3–5]. However, mammography is able to detect only 47~59 % of the underlying neoplasms confirmed by histology [3, 11, 12]; one-third of PD patients had an invasive cancer without a palpable mass or mammographic finding [5]. On the other hand, magnetic resonance imaging (MRI) is able to reveal the presence of 78~98 % of underlying cancers, and Amano et al. has suggested that MRI is very useful to assess the extent of these cancers accurately [3, 13].

Some investigators have reported that local recurrence is possible in patients with PD. One possible cause of recurrence may be insufficient preoperative evaluation of the lateral and vertical extent of the disease. In the present case, the extent of malignancy in the resected specimen was inconsistent with the gross changes of skin condition. Intraoperative consultation may be helpful for confirmation of the negative resection stump. For example, in patients with extra-mammary PD, mapping biopsy is often used preoperatively to evaluate dermal infiltration, and a 1-cm margin resection is acceptable [14–16].

Sentinel lymph node biopsy (SLNB) has been used as a standard option in clinically node-negative mammary cancer although the effectiveness of the use of SLNB for treatment of mammary PD has not been established. Laronga et al. published a database review comparing 36 patients with mammary PD who underwent SLNB and 18 patients who did not undergo SLN; 7 patients had sentinel lymph node metastases, all of which were associated with an underlying carcinoma. However, overall and disease-free survival did not differ significantly between the groups [17]. Two other reports have shown that not only patients with underlying invasive carcinoma had sentinel lymph node metastases but also patients without clinical evidence of an underlying carcinoma [18]. Therefore, because the reduced invasiveness of SLNB, this procedure was worth considering in the present case regardless of findings suggestive of an underlying carcinoma.

Although almost all the studies above have been with female mammary PD patients, given the reduced invasiveness of MRI, mapping biopsy, and SLNB, we suggest the use of these procedures for male patients with mammary PD to accurately assess the extent of the tumor.

## Consent

Written informed consent was obtained from the patient for publication of this case report and any accompanying images. A copy of the written consent is available for review by the Editor-in-Chief of this journal.

## Abbreviations

PD: Paget’s disease
MRI: magnetic resonance image
SLNB: sentinel lymph node biopsy
